# Automatic Quantitative Assessment of Lens Opacities Using Two Anterior Segment Imaging Techniques: Correlation with Functional and Surgical Metrics

**DOI:** 10.3390/diagnostics12102406

**Published:** 2022-10-04

**Authors:** Lars H. B. Mackenbrock, Grzegorz Łabuz, Timur M. Yildirim, Gerd U. Auffarth, Ramin Khoramnia

**Affiliations:** Department of Ophthalmology, University Hospital Heidelberg, 69120 Heidelberg, Germany

**Keywords:** anterior segment OCT, Scheimpflug imaging, cataract

## Abstract

The purpose of this study is to quantitatively assess lens opacity, using a swept-source optical coherence tomography (SS-OCT) device for anterior segment assessment, and establish the correlation with Scheimpflug imaging, corrected distance visual acuity (CDVA) and cumulative dissipated energy (CDE). This prospective cross-sectional single-center study enrolled 51 patients (51 eyes) with crystalline lens opacity. Patients with previous ocular surgery, pathologies or general disorders affecting vision were excluded. Eyes were scanned with an SS-OCT device, and lens densitometry was automatically analyzed using a custom MATLAB script which examined lens density, nuclear density and linear density. The same analyses were performed on Scheimpflug images. Preoperative CDVA and CDE during phacoemulsification were recorded. Spearman’s (ρ) and Pearson’s (r) correlation coefficients were assessed according to data normality. Statistically significant correlations were established between SS-OCT and Scheimpflug imaging using lens analysis (ρ = 0.47, *p* < 0.001), nuclear analysis (ρ = 0.73, *p* < 0.001) and linear analysis (r = 0.44, *p* < 0.001). A significant correlation with CDE was found with all the SS-OCT methods (r = 0.57, *p* < 0.001). Only the nuclear analysis of the SS-OCT scans (T_b_ = −0.33, *p* < 0.01) and Pentacam Nucleus Staging (T_b_ = −0.26, *p* < 0.05) showed a statistically significant correlation with CDVA. Good inter-device agreement in lens densitometry was found. However, SS-OCT yielded improved lens imaging compared with the Scheimpflug device and a higher correlation with clinical parameters. Thus, high-resolution SS-OCT has the potential to become a preferable option for automatic cataract grading and preoperative planning.

## 1. Introduction

Although the rate of cataract surgery is increasing, cataracts remain the leading cause of temporary reduction in vision which, if it is both the only ailment and properly managed, can be restored [1]. The developments of novel diagnostics and surgical techniques have improved the prediction accuracy of refractive outcomes and set patients’ expectations higher [2]. The need for a robust cataract-grading system becomes essential in decision making before surgery in order to achieve a desired clinical result and lower the complication rate [3]. To this date, the subjective Lens Opacities Classification System III (LOCS III) remains the gold standard in cataract grading [4]. However, it has been repeatedly shown that this method suffers from poor intra-observer reliability and that it is affected by slit-lamp settings [5,6]. A quantitative grading system has the potential to overcome these limitations. Scheimpflug tomography was introduced in the early 2000s for corneal diagnostics, utilizing the Scheimpflug principle by rotating and tilting a camera lens to acquire focused cross-section images of the anterior segment. Meanwhile, anterior segment swept-source optical coherence tomography (SS-OCT) has been gaining traction in recent years, following the success of retinal OCT. Similar to ultrasound imaging, a high-resolution image of the anterior segment is formed by detecting the signal backscattered from different tissues using a near-infrared laser.

Both methods have been validated as tools for conducting automatic cataract-severity assessments and have demonstrated good reliability in previous studies [7,8,9,10,11,12,13]. Previous authors, such as Chen et al., performed a manual nucleus densitometry analysis with an IOL Master 700, while Panthier et al. performed an automatic lens densitometry analysis using the same biometry device. Meanwhile, Makhotkina et al. measured the nuclear density manually by taking into account only a fraction of the lens. These studies found a correlation between SS-OCT, Scheimpflug imaging and LOCS III, as well as corrected distance visual acuity (CDVA) and cumulative dissipated energy (CDE) [10,14,15]. However, these previous works comparing SS-OCT and Scheimpflug tomography suffer either from a small sample size, limited lens density analysis methods or using non-automatic image analysis, as well as using older SS-OCT devices with reduced spatial resolution.

It has been shown that through the creation of hydroxyradicals, high levels of CDE can result in a longer post-operative recovery time due to endothelial cell loss or pupillary impairment, particularly in eyes suffering from corneal conditions such as Fuchs’ endothelial dystrophy [16,17,18,19]. Therefore, by establishing a cut-off point where phacoemulsification induces functionally significant endothelial loss, an automatic grading system could facilitate determining the appropriate time for cataract surgery, thus reducing the risk of potential ocular trauma due to higher levels of CDE. By quantifying factors that reduce visual acuity linked to lens opacity, such as light scattering, refractive error and higher-order aberrations, it would be possible to provide an estimate of post-operative visual function.

The aim of this study was to determine the correlation between the established Pentacam AXL Wave (Scheimpflug technology; OCULUS Optikgeräte GmbH, Wetzlar, Germany) and the novel ANTERION (SS-OCT technology, Heidelberg Engineering GmbH, Heidelberg, Germany) in terms of automatic quantitative lens density evaluation, as well as to determine how these measurements correlate with CDE and CDVA using different opacity analysis methods.

## 2. Materials and Methods

### 2.1. Patients

Fifty-one eyes from fifty-one patients scheduled to undergo routine cataract surgery at the Heidelberg University Hospital (Heidelberg, Germany) between September 2021 and January 2022 were enrolled in this prospective study. Adult patients with senile, traumatic or iatrogenic cataracts were included. No patient had a history of previous ocular surgery, an ocular condition (such as uveitis, glaucoma, retinal disorder, central cornea opacities, etc.) or systemic disorders affecting vision. Patients with poor fixation, a spherical equivalent of ±8 dpt, as well as mydriasis <5 mm were also excluded. The initial study population consisted of 70 patients, of whom 19 were excluded, as they either decided not to undergo surgery (10), had insufficient mydriasis (6) or demonstrated inadequate compliance (3). Only one eye was evaluated in this study. If both eyes required surgery, the one with the best image quality was selected. This study was conducted in accordance with the Declaration of Helsinki upon obtaining written informed consent from each participant. The preoperative examination included Snellen CDVA, slit-lamp microscopy, Goldman applanation tonometry and a comprehensive fundus examination, with an additional OCT screening of the macula.

### 2.2. Devices

ANTERION (Heidelberg Engineering GmbH, Heidelberg, Germany) is an SS-OCT device that utilizes a 1310 nm swept-source laser to produce high-resolution images and analysis of the anterior segment. It has an A-Scan rate of 50,000 Hz and uses up to 1024 A-Scans per B-Scan. Eye tracking allows for the possibility to average up to 8 B-Scans to increase image quality. It provides an axial resolution of <10 μm and a lateral resolution of 30 μm. This system has not yet been used to assess lens densities. Full mydriasis was achieved in all patients with phenylephrine 5% (URSAPHARM Arzneimittel GmbH, Saarbrucken, Germany) and tropicamide (Pharma Stulln GmbH, Stulln, Germany) eye drops. Patients were instructed to stabilize their gaze on the fixation point and to keep the eye wide open during the scan to avoid shadowing artifacts from the eyelids. All examinations were performed by a single experienced operator. A radial pattern centered on the optical axis, consisting of 15 B-Scans of 14 mm length, was used to produce high-resolution cross-sections of the anterior segment. Images that did not meet the build-in quality criteria due to blinking, bad alignment or poor fixation were excluded, and the examination was repeated.

Pentacam AXL Wave (OCULUS Optikgeräte GmbH, Wetzlar, Germany) uses a CMOS sensor and a 475 nm UV-free blue LED to capture up to 100 Scheimpflug images in a radial pattern of the anterior segment in 2 s. The Scheimpflug examination was performed directly after the SS-OCT scan to ensure similar conditions. The protocol was identical to the SS-OCT scan and was performed by the same operator in complete darkness. Scans that failed the quality check were discarded and repeated. The measurements were also repeated if the images contained artifacts which could interfere with the densitometric analysis, such as irregular illumination, blinking or insufficient mydriasis.

### 2.3. Surgical Technique

All patients underwent uneventful cataract surgery performed by two experienced surgeons (R.K. and G.U.A.). Phacoemulsification was performed using the same technique with a Centurion Vision System (Alcon, Geneva, Switzerland). As the use of a femtosecond laser to perform capsulotomy and lens prefragmentation reduces CDE [20], patients were divided into two groups, where one was operated with the laser (LensX Lasers Inc., Aliso Viejo, CA, USA) and the other without it. CDE is displayed automatically by a Centurion Vision System and is traditionally calculated as CDE (%s) = Average phacoemulsification power (%) x Phacoemulsification time (s) [21]. The patient group operated with the femtosecond laser was excluded from the CDE analysis.

### 2.4. Image Analysis

**Figure 1:**
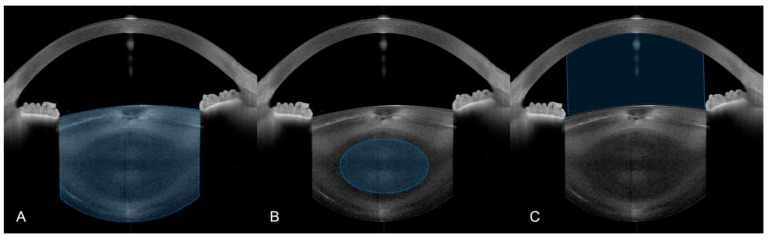
Automatic segmentation of (**A**) the entire lens, (**B**) the nucleus and (**C**) the anterior chamber in SS-OCT images performed by a custom MATLAB script.

Three distinct analyses of the SS-OCT data were performed: lenticular, nuclear and linear opacity. A custom MATLAB (Version R2021b, MathWorks, Natick, MA, USA) script automatically calculated the lens opacity using a method briefly discussed here: After contrast adjustment, images were binarized using a general threshold. Following noise removal and a morphological closing operation, a clean binary image was obtained. The approximate cross-sectional shape of the anterior and posterior lens surface resembles the curve described by a polynomial of the 4th order, which can be used to correct local segmentation errors. The script computed the 4th-order polynomial that best fit the detected edge and corrected points that substantially deviated from this curve. The mean pixel intensity (on a scale of 0 to 255) of the segmented area represents the lens opacity (Figure 1A). Following the method established by Wong et al. to compensate for background noise [15], the corrected lens opacity was calculated by subtracting the mean pixel intensity value of the anterior chamber from the lens opacity. Anterior chamber segmentation was performed by implementing Canny edge detection to detect the borders of the cornea using the anterior surface of the lens to create a concave/convex region of interest (ROI) (Figure 1C) [22]. To counteract pixel intensity variations between individual B-Scans due to anatomic differences and uneven illumination, the final lens density was derived by averaging all 15 B-Scans.

To calculate the nuclear opacity, the script positioned an elliptical ROI at the center of the previously segmented lens (Figure 1B). Again, all 15 B-Scans were averaged, and the background noise was subtracted to obtain the nucleus opacity value. All segmentations were reviewed by two independent and experienced observers to detect and manually correct any errors produced by the automatic segmentation, which, of 765 images, was only necessary for 3, indicating the robustness of this approach.

**Figure 2:**
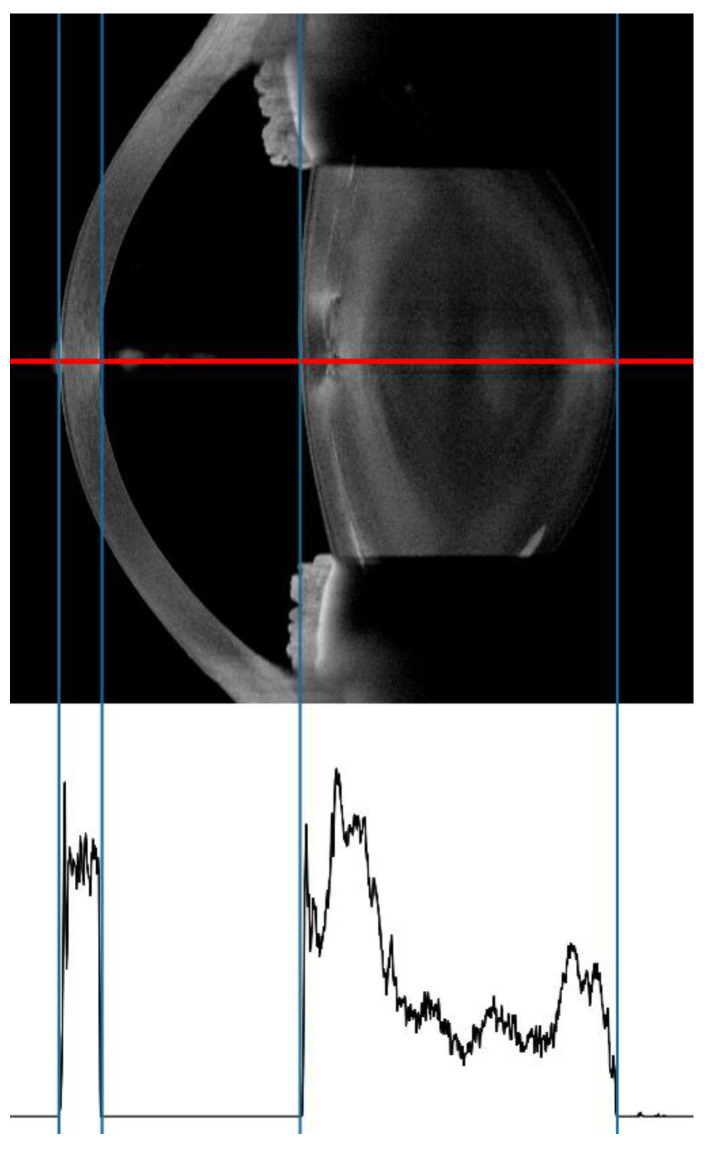
Linear density analysis using SS-OCT. The graph represents the intensity values along the red line (A-Scan). Linear density is calculated through the mean of the values between the 3rd and 4th blue line.

To obtain the linear opacity, the raw intensity values produced by the biometry software of the SS-OCT device were exported. These represent the intensity values of the A-Scan along the optical axis, using the device’s arbitrary units which, according to the manufacturer, are normalized by the dynamic range of its detector. The MATLAB script automatically detected the intensity range representing the lens and calculated the mean intensity between the anterior and posterior capsule (Figure 2).

**Figure 3:**
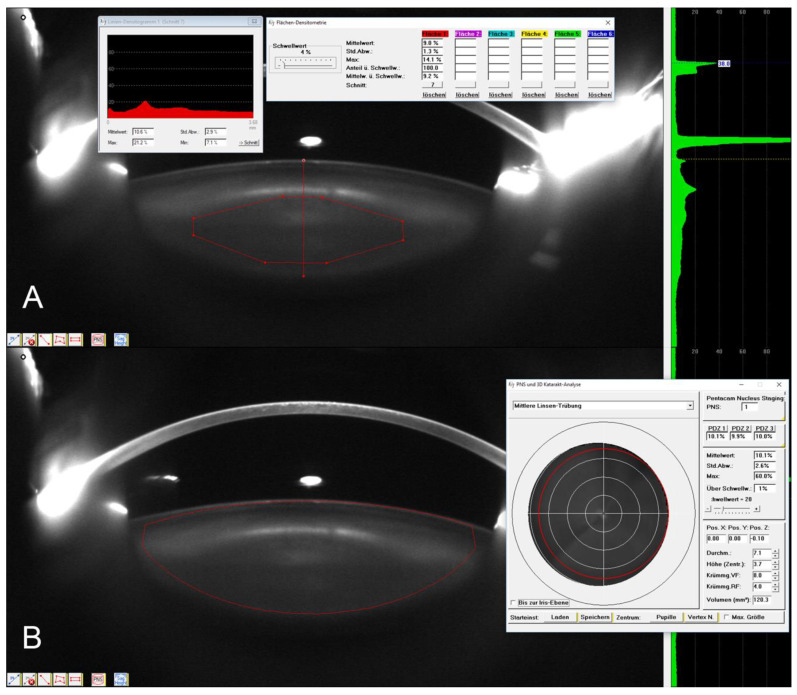
Manual densitometric analysis of the Pentacam images using the nucleus (ROI) (**A**), the linear method (**A**) and the lens (3D) method (**B**).

Four distinct densitometric analyses were performed on the Scheimpflug images using methods established in previous studies: linear, ROI, three-dimensional (3D) and Pentacam Nucleus Staging (PNS) [10]. In the linear method, a line is drawn along the optical axis through the lens, while in the ROI method, an octagonal ROI is drawn inside the nucleus (Figure 3A). The Pentacam software automatically calculates the average density of these geometric shapes, where 0% represents ocular-media clarity and 100% complete lenticular opacity. The nucleus occupied on average 14% of the lens area with both SS-OCT and Scheimpflug imaging. We only selected the Scheimpflug images on, or next to, the vertical axis (90–270°) to ensure comparability between the patients. As opposed to the linear and ROI methods, the 3D method calculates the average lens density by manually placing an elliptical ROI encompassing the nucleus and cortex (Figure 3B), which is then automatically applied to all images, creating an ellipsoid volume. Each ROI placement was performed by a single examiner and reviewed by an independent observer. A high repeatability of manual lens density assessment with Scheimpflug imaging has been confirmed in the literature [13,14]. While all these methods rely on manual input, PNS automatically grades the cataracts on an ordinal scale from 0 to 5, taking into account all images taken by the Pentacam.

### 2.5. Statistical Analysis

All results were analyzed using SPSS (Version 28, IBM, Chicago, USA). Values are reported as means ± standard deviation. Data normality was assessed using the Kolmogorov–Smirnov test. As data normality was not provided for all parameters, the Pearson (r) or Spearman (ρ) correlation coefficients were used accordingly to analyze the different methods. Kendall’s tau-b was used to analyze non-normally distributed data and discrete variables. A *p*-value < 0.05 was considered significant. A sample size calculation showed that, assuming a true correlation between the lens opacity measurements of the two devices of 0.4, a sample size of 37 would have a power of 90% to detect a Pearson’s correlation coefficient different from 0, using a two-sided test at a significance level of 0.05.

## 3. Results

Fifty-one eyes of fifty-one patients (27 women and 24 men) with an average age of 63.2 ± 10.2 years were examined in this study. The mean age of the female patients was 61 ± 12 years, and the mean age of the male patients was 65 ± 7 years. As only one eye was selected, the dataset consisted of 32 right eyes and 19 left eyes.

**Figure 4:**
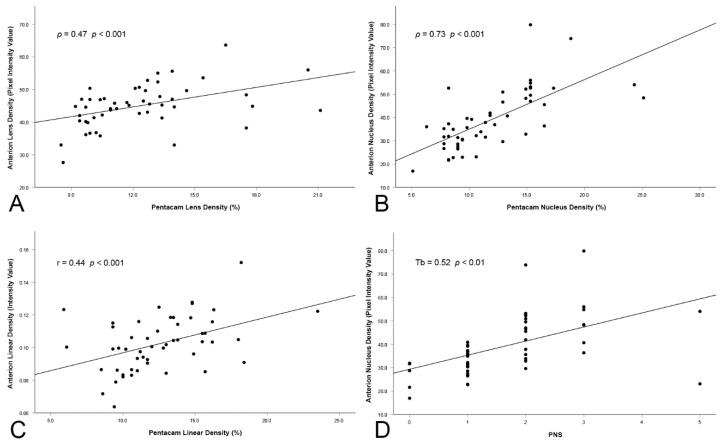
Scatter plots of the correlations between SS-OCT and Scheimpflug imaging using the (**A**) lens method, (**B**) nucleus method and (**C**) linear method. (**D**) is the correlation between PNS and the nucleus method used on the SS-OCT scans.

The densitometric results for both devices are outlined in Table 1, while the correlation coefficients are given in Table 2. While the highest correlation coefficient was found when comparing the nucleus (ρ = 0.73 with *p* < 0.001), there were statistically significant correlations between both devices, regardless of the method used. An overview of the most relevant comparisons can be found in Figure 4.

**Figure 5:**
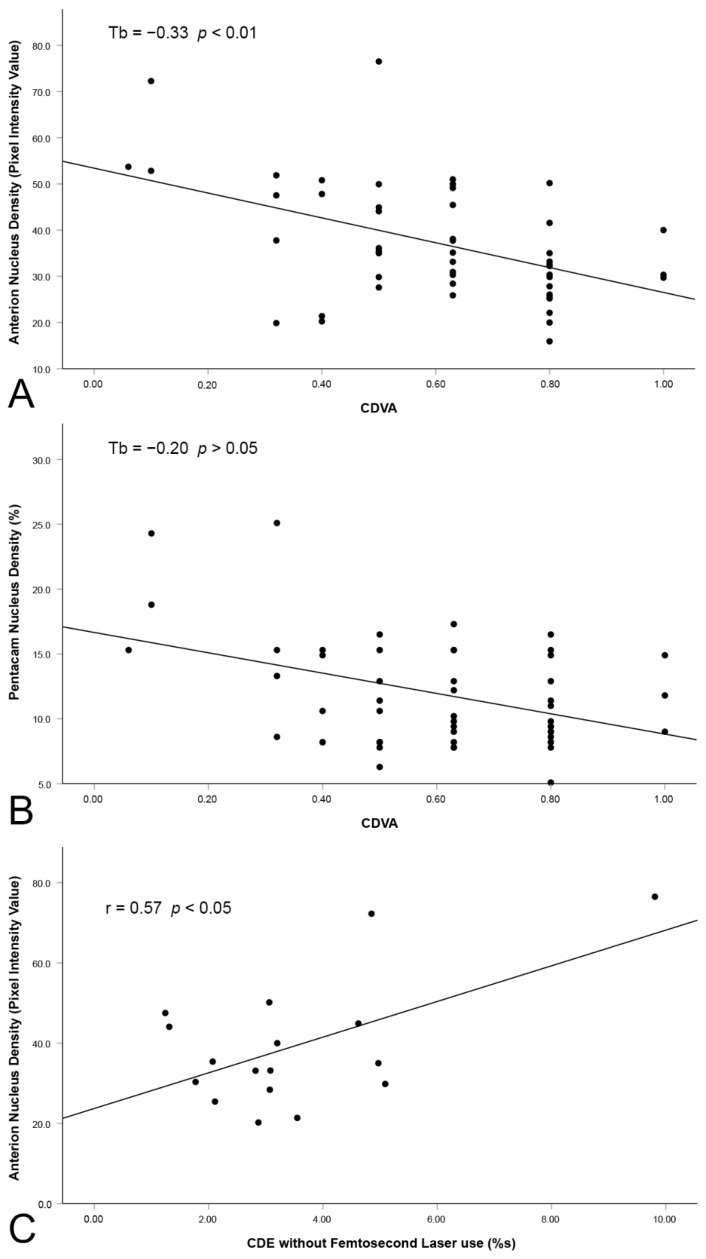
Scatter plots of the correlation between (**A**) nuclear density in SS-OCT and CDVA, (**B**) nuclear density in Scheimpflug imaging and CDVA, and (**C**) lens density in SS-OCT and CDE without femtosecond laser use.

Preoperative Snellen CDVA was, on average, 0.57 ± 2.4 lines. The correlation with the most interesting lens density analysis methods can be found in Figure 5, where the highest correlation was achieved with the nucleus method applied to the SS-OCT scans (T_b_ = −0.33 with *p* < 0.01).

The CDE data have been documented for 25 patients in this study, with the applied ultrasonic energy ranging from 1.24 %s to 9.81 %s, with a mean of 3.26 ± 1.79 %s. A statistically significant correlation (Table 2) was found with all the SS-OCT analysis methods (the nucleus method being, again, the most important), as well as with the linear method with the Scheimpflug imaging device (r = 0.57 with *p* < 0.001).

## 4. Discussion

A reliable quantitative lens opacity assessment method could help prevent intra- and post-operative complications in routine cataract surgery, as well as provide a benchmark for clinical studies. The ability to precisely determine cataract severity gives surgeons the choice to adapt phacoemulsification settings accordingly, which could lead to more efficient and safer surgeries with faster recovery times and better outcomes [23].

The aim of this study was to establish the correlation between two devices utilizing different imaging techniques, as well as CDVA and CDE, and to explore the impact each approach may have. On the one hand, the SS-OCT scans were automatically analyzed, manually reviewed and corrected wherever necessary using a custom MATLAB script, which ensured the highest repeatability while making it possible to quickly grade a large number of cataracts. The Scheimpflug images, on the other hand, were manually analyzed using the build-in software due to the lack of a raw-image export option.

Chen et al. found a significant correlation between Scheimpflug imaging and SS-OCT scans from an IOL Master 700 (Carl Zeiss Meditec AG, Jena, Germany) when analyzing the nucleus [9]. Panthier et al. also compared SS-OCT scans from an IOL Master 700 using a manual and automatic method to analyze the entire lens [24,25]. Both methods yielded a higher correlation with the PNS score than reported here (r^2^ = 0.75, compared to our T_b_ = 0.52). Both study groups used the same swept-source OCT device and obtained a higher correlation with Scheimpflug imaging. Therefore, how the backscattering produced by IOL Master 700 differentiates from ANTERION backscattering should be explored in a future study.

Makhotkina et al. found a less important correlation coefficient between SS-OCT and different Scheimpflug imaging density measurements (ρ = 0.49, compared to our r = 0.73) [26], which might be due to their analysis method: they placed a small rectangular ROI in the posterior half of the nucleus in SS-OCT images. However, cataracts are not always homogeneously dispersed throughout the nucleus; therefore, this method is prone to overlooking certain irregular opacities [27]. This could also explain their lower correlation with CDE compared to the current study (ρ = 0.41, compared to our r = 0.57).

Overall, SS-OCT performed better than Scheimpflug imaging when establishing the correlation between CDE and CDVA in our study. Particularly, nuclear density in the SS-OCT scans correlated better with CDVA than the Pentacam images (T_b_ = −0.33 *p* < 0.01 vs. ρ = −0.27 *p* = 0.061), which is in line with the results of Bourdon et al. Although statistically significant, the correlation we established was low, which might have been due to the difference in scales. We observed the highest correlation between CDE and cataract assessment when considering the nucleus with SS-OCT [7], followed closely by the lenticular density. Heyworth et al. concluded that lens hardness comes primarily from the nucleus [28]; therefore, it is reasonable that cortical cataracts do not influence the CDE, which has been established by Bencić et al. and is in line with our findings [29]. It should be noted that while the CDE is an indicator for lens hardness, it remains sensitive to numerous factors, such as surgeon experience and preference [23]. The moderate correlation value (r = 0.57) is in part due to the comparison of the objective assessment of lens density against the subjective use of phacoemulsification.

The goal of zero-phacoemulsification surgery is becoming a reality for ever more patients thanks to new surgical devices such as the femtosecond laser [30]. While intraoperative ultrasonic energy is generally considered safe, it can lead to clinically relevant endothelial cell loss in patients suffering from corneal conditions [17]. Therefore, determining a time point to perform phacoemulsification in which the lens is still soft and the potential for ultrasound-related trauma to the ocular tissue remains low is certainly of interest. Two patients in the current study underwent femtosecond laser-assisted zero-phacoemulsification surgery. Both had the lowest nuclear density of the cohort (15.92 Pixel Intensity Units and 16.00 PIU measured with the SS-OCT device). A cut-off point for zero-phacoemulsification based on an automated grading approach should be the subject of future research. Such an objective measure could help clinicians decide whether surgery could be postponed without risk to a patient.

In addition to a higher resolution of the SS-OCT device, which may contribute to improved imaging, the use of 1310 nm by ANTERION instead of 475 nm (Pentacam) may be another important factor. Boettner and Wolter demonstrated a higher light transmission of the cornea in the infrared range than in shorter wavelengths, which may hamper the visualization of the lenticular area [31]. As a result, it can be challenging to detect the posterior capsule in Scheimpflug imaging, a limitation also reported by Chen et al. [9]. The thickest lens that was successfully imaged using the SS-OCT device was 6.39 mm in diameter with a total ocular depth of 11.23 mm, while with Scheimpflug imaging the ocular depth was 7.25 mm, but only 3.76 mm of the lens was visible. However, one should consider that maximum imaging depth depends on numerous factors such as lens opacity, pupil size, tear film quality and number of B-Scan averaging; hence, an absolute value cannot be determined. It should also be noted that the Pentacams’ primary objective is corneal diagnostics [32,33,34,35]. Therefore, one may expect that an SS-OCT device enables a better view of the entire lens thickness, making it difficult to compare the lenticular opacity between both systems. The lack of a clear posterior edge could also lead to user bias during the manual lens segmentation, which may explain the weakest correlation when using the linear method (r = 0.44 with *p* < 0.001) [13]. While it has the advantage of less data processing, the literature points out its poor reliability [10,14].

Another drawback of using Scheimpflug imaging is the need for maximum mydriasis, while Li et al. concluded that mydriasis was not necessary for reliable lens assessment using SS-OCT [36]. As a result, SS-OCT has the potential to provide an enhanced cataract grading tool, which is also supported by this study’s results. A clinical application of the proposed methods in the preoperative planning and management of patients scheduled for cataract surgery requires further research.

One limitation of the current study is that we were not able to record the CDE for all patients. Additionally, excluding the patients where the femtosecond laser was used, to avoid confounding our data, resulted in a relatively small sample size. Although the LOCS III grading system was not applied to classify the patients’ cataracts, the studied approach has already been validated and has shown a good correlation with the LOCS III score in previous studies [8,10,11,12,15]. While Chen et al. reported that SS-OCT correlates better than Scheimpflug imaging with LOCS III NO and NC [9], Makhotkina and co-workers did not confirm this [26]. Given that factors such as ROI location or physician experience may impact the results, a fully automated approach is desirable.

Contrast sensitivity was shown to suffer with the progression of cataracts due to light scattering and spherical aberrations, significantly reducing visual function [37]. Grewal et al. demonstrated that contrast sensitivity correlated with Pentacam nuclear density [38]. How the proposed method of automatic lens opacity grading correlates with this loss of contrast sensitivity should be explored in a future study. SS-OCT, which relies on backscattering, supported by a device that measures forward light scatter, may improve such assessment.

Ultrasound biomicroscopy (UBM) has gained traction as a tool for congenital cataract evaluation where lens opacity characteristics are more complex than in senile cataracts [39]. While the ability of UBM to assess structures situated behind the iris is certainly of interest, its dependence on operator skill and a lower resolution could limit its use in systematic cataract grading. For instance, when compared to SS-OCT, Tabatabaei et al. demonstrated that UBM was less suited for traumatic cataract surgical planning [40].

In conclusion, this study demonstrated a statistically significant inter-device correlation in the lens densitometry analysis between ANTERION and Pentacam. However, our data suggest that SS-OCT may predict CDVA and CDE better than Scheimpflug imaging, giving it an edge as a clinical diagnostics device. Novel neural network-based segmentation image-processing procedures will refine these methods by enabling the precise quantifications of nuclear, cortical and capsular opacities [41,42]. When used in combination with the femtosecond laser, it could help pave the way towards more zero-phacoemulsification surgeries, reducing the strain on ocular structures and improving post-operative recovery times, particularly with patients already suffering from ocular conditions.

## Figures and Tables

**Table 1:** Overview of the data provided by the different densitometric analysis methods applied to the ANTERION and Pentacam Wave AXL scans.

Device	Region of Interest	Range	Mean
ANTERION (Pixel Intensity Value)	Lens	27.59–63.66	44.78 ± 6.68
	Nucleus	15.92–79.84	39.08 ± 12.91
	Linear	0.064–0.153	0.102 ± 0.017
Pentacam (%)	Lens	8.5–21.1	12.3 ± 2.9
	Nucleus	5.1–25.1	11.9 ± 4.1
	Linear	5.9–23.5	12.5 ± 3.3

**Table 2:** Spearman, Pearson (_r_) or Kendall’s tau-b (_t_) correlations of lens density measurements, cumulative dissipated energy and corrected distance visual acuity. Only the correlation with CDE values without femtosecond laser use are displayed.

	Pentacam				ANTERION				
	3D (Lens)	Nucleus	Linear	PNS ^c^	Lens	Nucleus	Linear	CDE ^a^	CDVA ^b^
**Pentacam**									
3D (Lens)		0.77 ***	0.78 ***	0.81 _t_ **	0.47 ***	0.57 ***	0.49 ***	0.13	−0.16 _t_
Nucleus			0.74 ***	0.67 _t_ **	0.45 ***	0.73 ***	0.54 ***	0.18	−0.20 _t_
Linear				0.61 _t_ **	0.46 ***	0.48 _r_ ***	0.44 _r_ ***	0.57 _r_ *	0.03 _t_
PNS ^c^					0.39 _t_ **	0.52 _t_ **	0.46 _t_ **	0.21 _t_	−0.26 _t_ *
**ANTERION**									
Lens						0.78 _r_ ***	0.91 _r_ ***	0.53 _r_ *	−0.19 _t_
Nucleus							0.80 _r_ ***	0.57 _r_ ***	−0.33 _t_ **
Linear								0.50 _r_ *	−0.22 _t_ *
CDE ^a^									−0.02 _t_

^a^ Cumulative dissipated energy. ^b^ Corrected distance visual acuity. ^c^ Pentacam Nucleus Staging. * *p* < 0.05, ** *p* < 0.01, *** *p* < 0.001.

## Data Availability

All available data generated or analyzed during this study are included in this published article.
